# Major drivers of healthcare system costs and cost variability for routine atrial fibrillation ablation

**DOI:** 10.1016/j.hroo.2022.12.014

**Published:** 2022-12-27

**Authors:** Brian Zenger, Haojia Li, T. Jared Bunch, Candice Crawford, James C. Fang, Christopher A. Groh, Rachel Hess, Leenhapong Navaravong, Ravi Ranjan, Jeff Young, Yue Zhang, Benjamin A. Steinberg

**Affiliations:** ∗ University of Utah School of Medicine, Salt Lake City, Utah; † Department of Internal Medicine, Division of Epidemiology, University of Utah, Salt Lake City, Utah; ‡ Division of Cardiovascular Medicine, Department of Internal Medicine, University of Utah, Salt Lake City, Utah; § Decision Support, University of Utah Health, Salt Lake City, Utah; ¶ Division of General Internal Medicine, Department of Internal Medicine, University of Utah, Salt Lake City, Utah; || Department of Population Health Sciences, University of Utah, Salt Lake City, Utah

**Keywords:** Atrial fibrillation, Ablation, Costs, Value-driven healthcare, Provider preferences, Cost variability

## Abstract

**Background:**

Catheter ablation is an effective treatment for atrial fibrillation (AF) but incurs significant financial costs to payers. Reducing variability may improve cost effectiveness.

**Objectives:**

We aimed to measure (1) the components of direct and indirect costs for routine AF ablation procedures, (2) the variability of those costs, and (3) the main factors driving ablation cost variability.

**Methods:**

Using data from the University of Utah Health Value Driven Outcomes system, we were able to measure direct, inflation-adjusted costs of uncomplicated, routine AF ablation to the healthcare system. Direct costs were considered costs incurred by pharmacy, disposable supplies, patient labs, implants, and other services categories (primarily anesthesia support) and indirect costs were considered within imaging, facility, and electrophysiology lab management categories.

**Results:**

A total of 910 patients with 1060 outpatient ablation encounters were included from January 1, 2013, to December 31, 2020. Disposable supplies accounted for the largest component of cost with 44.8 ± 9.7%, followed by other services (primarily anesthesia support) with 30.4 ± 7.7% and facility costs with 16.1 ± 5.6%; pharmacy, imaging, and implant costs each contributed <5%. Direct costs were larger than indirect costs (82.4 ± 5.6% vs 17.6 ± 5.6%). Multivariable regression showed that procedure operator was the primary factor associated with AF ablation overall cost (up to 12% differences depending on operator).

**Conclusions:**

Direct costs and other services (primarily anesthesia) drive the majority costs associated with AF ablations. There is significant variability in costs for these routine, uncomplicated AF ablation procedures. The procedure operator, and not patient characteristic, is the main driver for cost variability.

**Key Findings**

▪ Direct costs, including electrophysiology lab supply costs and other services with anesthesia, make up the bulk of healthcare system atrial fibrillation ablation costs.
▪ The cost variability, even in routine outpatient atrial fibrillation ablation procedures, is large.
▪ Procedure operator was the main factor associated with cost variability, yielding a 12% difference in atrial fibrillation ablation costs between the least and most expensive operators.

## Introduction

Atrial fibrillation (AF) is the most common sustained arrhythmia in clinical practice, and the cost of treatment for the arrhythmia and its comorbidities is a significant burden on the healthcare system.^1^ Furthermore, the prevalence of AF is expected to increase to over 12.1 million people in the United States by the year 2030, driven by an aging population with more cardiovascular comorbidities that will continue to increase and further stress the healthcare system.^2^ Though expensive, studies of payer costs have demonstrated favorable cost-effectiveness of AF ablation, based on lower long-term management costs compared with medical therapy alone.^1^^,^^3^^,^^4^ Nevertheless, there is significant variability in healthcare system payments for AF ablation, without a clear understanding of factors that drive cost, or variability in costs, to healthcare systems delivering these procedures.^3^

Quantifying costs of care delivery for a healthcare system is vital to improving value and to containing overall costs within a financially constrained environment. Reducing variability can be an effective means to reducing costs and improving efficiency. Communicating to clinicians the cost components has been shown reduce overall costs of care and to increase both patient perceived and objective metrics of quality, particularly related to medical procedures.^5^ In this study, we utilized the singular, Value Driven Outcomes system developed at University of Utah Health to assess the variability of healthcare system costs for AF ablation procedures. There were 3 specific objectives of our study: (1) define the components that drive the direct and indirect costs of AF ablation procedures to a healthcare system, (2) describe the variability in AF ablation costs to the healthcare system, and (3) identify the primary factors associated with variability in AF ablation costs.

## Methods

### Cost data collection

In 2013, the University of Utah developed the Value Driven Outcomes system, which compiles and tabulates all components of a healthcare encounter that contribute to the cost of delivering that care for the healthcare system: physician compensation, facility resources, supplies, ancillary services, pharmacy, imaging, and laboratory tests, among others.^5^, ^6^, ^7^, ^8^, ^9^ Details of this unique program have been previously described.^5^ In brief, aggregate costs, such as building space, equipment, labor, and professional services, are approximated based on a patient’s estimated, pro-rated use of such resources. Supplies, medications, and contracted service costs are based on the healthcare system’s actual purchase rate. For this analysis, we separated costs into 7 categories: facility (overwhelmingly staff labor), imaging, implant supplies (primarily vascular closure devices, where applicable), electrophysiology (EP) clinical lab management, pharmacy, other supplies (eg catheters and ablation equipment), and other services (primarily anesthesia services). Common disposable, AF ablation tools such as ablation catheters and consumables were under the category “other supplies.” Costs were then separated into direct and indirect costs to the medical system. Given the outpatient nature of the included procedures, anesthesia support was the primary component of “other services.” Because of their primary contributions, we refer to “other services” as anesthesia support and “other supplies” as ablation equipment to avoid confusion. Direct costs were identified as pharmacy, supply, patient lab, implant, and anesthesia support. Indirect costs were identified as facility, imaging, and lab management costs. Only costs related to the ablation procedure encounter were used in this analysis, and no postdischarge costs were included if they were part of separate admissions or encounters. However, any postprocedure care within the same encounter (eg, next day prior to discharge), such as imaging, medications, or laboratory tests, were included. All absolute cost values were inflation corrected using the using the monthly Consumer Price Index for All Urban Consumers healthcare inflation cost index. As the objectives centered around variability of routine, uncomplicated ablation procedures were defined as (1) planned outpatient procedures and (2) those performed by an operator with 10 or more AF ablations performed during the examined time period. Procedures were excluded if (1) they were not outpatient status, (2) they were performed by an operator with <10 total cases during the study period, (3) the patient length of stay was >1 night, or (4) if the total cost of the procedure was below a minimally feasible amount for the procedure.

Due to the sensitive and competitive nature of absolute cost values, the institution implements guidelines for their public reporting. As such, data reported herein do not include absolute cost numbers, or components that could be used to derive such numbers (see Statistical Methods).

### Study population

Patient cost data for AF ablation procedures performed at the University of Utah were collected from January 1, 2015, to December 31, 2020, as defined by Current Procedural Terminology code 93656. Clinical data are derived from the healthcare system’s enterprise data warehouse and include all administrative billing encounters with diagnosis codes (inpatient, outpatient, procedural), as well as medication orders, laboratory results, electrocardiography results, and echocardiography results, according to previously described methodology.^10^^,^^11^ Clinical comorbidities were measured using previously validated algorithms for use in administrative data analyses of cardiovascular disease and included all healthcare system encounters up to and including the index visit. Comorbidity rates were calculated based on International Classification of Diseases codes as part of clinical billing encounters, as previously described.^10^^,^^12^

### Statistical methods

The distribution of patients’ demographic characteristics and medical conditions were summarized for both numeric and categorical variables using descriptive statistics. We also employed bar and histogram plots to summarize the composition of total cost by major cost components and distribution of cost in each major component. We used the variance inflation factor to detect if multicollinearity is present among the list of potential driving factors. Because none of potential driving factors has a variance inflation factor exceeding 4, we treated all factors as independent or mildly correlated and performed multivariate generalized linear regression with the least absolute shrinkage and selection operator (LASSO) to identify the subset of potential predictors that are informative for predicting the total cost of AF ablation procedure. The final model for total cost was determined based on the optimal penalty term using 10-fold cross-validation criteria. By imposing some penalty in the regression model fitting, the LASSO approach can shrink the coefficients of those unimportant predictors to zero while retaining those important ones. Note that the LASSO approach selected predictor based on whether its coefficient is nonzero instead of *P* values. Thus the final models shall include all important predictors with parsimonious representation, enhanced interpretability, and improved prediction precision. From the soft-thresholding property of LASSO in the generalized linear regression models, the estimated regression coefficient is biased toward zero.^13^, ^14^, ^15^ To mitigate these bias problems, we reported a more unbiased estimation of the regression coefficients from unpenalized multivariate logistic regression using the selected factors in LASSO. Most of potential factors in the dataset had no missing values, except the left ventricular ejection fraction (LVEF) level variable. We used the missing indicator approach where we treated missing LVEF as an unobserved category for categorized LVEF (ie, normal, abnormal, and unobserved categories).

Data processing was performed using R (version 3.6.3; R Foundation for Statistical Computing, Vienna, Austria) and RStudio (version 1.2.5033; RStudio, Boston, MA), with appropriate packages. Statistical analysis was performed using R (version 4.1.0; R Foundation for Statistical Computing) and RStudio (version 1.0.153; RStudio).^12^^,^^16^ Analysis of the data collected as part of routine clinical care, and subsequent reporting of anonymized, aggregate data, was approved by the University of Utah Institutional Review Board. Consent was waived by the Institutional Review Board because the study is a retrospective analysis with minimal risk to patients. The research reported adheres to the Helsinki Declaration guidelines on human research.

## Results

A total of 910 patients with 1060 outpatient ablation encounters were conducted from January 1, 2015, to December 31, 2020 and met criteria for inclusion. Relevant baseline patient characteristics are found in Table 1.

**Table 1:** Baseline patient characteristics of patients included in this study (N = 910)

Age, y	

Mean ± SD	65.2 ± 11.33
Median (IQR)	67 (58.25, 73.00)
Range	24–92
Female	302 (33%)
White	860 (95%)
BMI, kg/m^2^	30.8 ± 6.7
Hypertension	469 (52%)
Diabetes mellitus	202 (22%)
Myocardial infarction	222 (24%)
Congestive heart failure	299 (33%)
Chronic kidney disease	108 (12%)
Peripheral vascular disease	278 (31%)
Pulmonary disease	278 (21%)
History of stroke	91 (10%)
CHA_2_DS_2_-VASc score	
Mean ± SD	2.83 ± 1.94
Median (IQR)	3 (1, 4)
Range	0–9
Prior cardioversion	349 (38%)
Prior ablation	204 (22%)
Anticoagulation	859 (94%)
Beta-blocker	587 (65%)
Calcium-channel blocker	266 (29%)
Antiarrhythmic drugs	638 (70%)
Left ventricular ejection fraction, %	
Mean ± SD	56.55 ± 11.32
Median (IQR)	60 (52.5, 63)
Range	15–80

Values are n (%) or mean ± SD, unless otherwise indicated.

BMI = body mass index; CHA_2_DS_2_-VASc = congestive heart failure, hypertension, age ≥75 years, diabetes mellitus, prior stroke or transient ischemic attack or thromboembolism, vascular disease, age 65-74 years, sex category; IQR = interquartile range.

### Cost data

**Figure 1:**
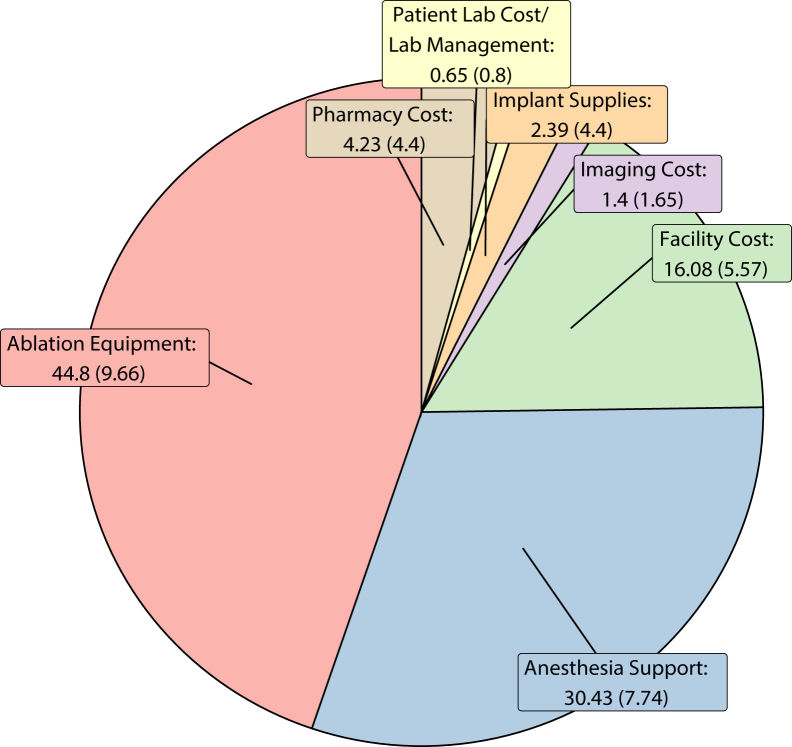
Pie chart of the average (standard deviation) percentage of cost comprised of each category investigated.

**Figure 2:**
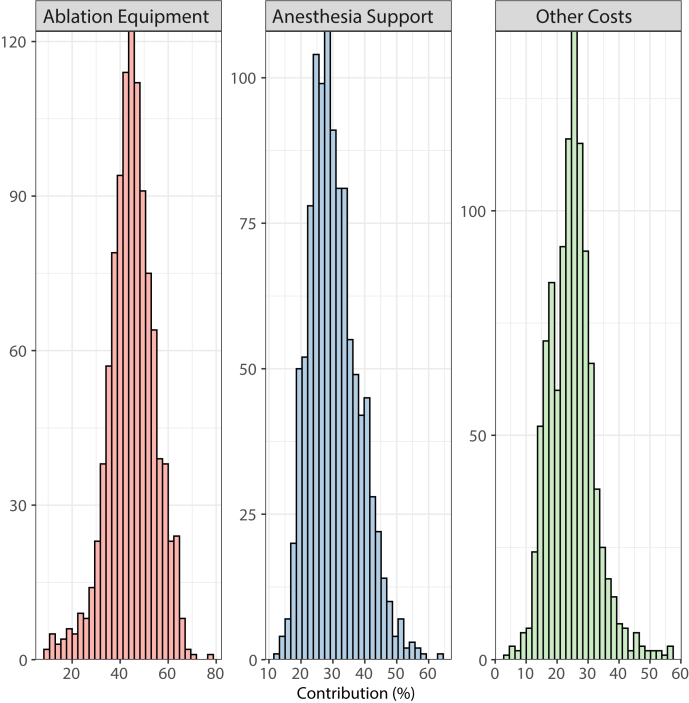
Histograms for proportion of anesthesia support, ablation equipment, and all other costs (all other costs defined as facility, implant, imaging, pharmacy, and electrophysiology lab management costs). Note that contribution percentages (x-axis) vary from 0% to 100% with frequency of occurrence (y-axis) could vary from 0 to 1060 ablation procedures.

Figure 1 shows the proportions of cost based across 7 categories. Overall cost of ablation equipment was the largest component of cost with 44.8 ± 9.7%, followed by anesthesia support with 30.4 ± 7.7% and facility costs with 16.1 ± 5.6%. Pharmacy, imaging, and implant supplies were all under 5% each. The distribution of proportions contributing to cost for major components are shown in Figure 2. Detailed histograms of each category are shown in Supplemental Figure 1.

### Direct vs indirect costs

**Figure 3:**
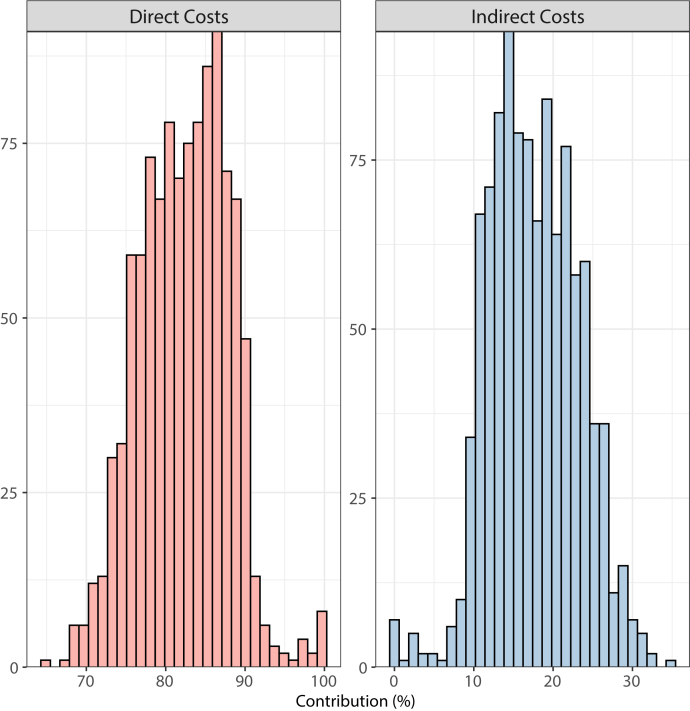
Histogram distribution of contribution of overall atrial fibrillation ablation costs of direct vs indirect costs. Note that contribution percentages (x-axis) vary from 0% to 100% with frequency of occurrence (y-axis) could vary from 0 to 1060 ablation procedures.

Overall direct costs were larger, with 82.4 ± 5.6%, compared with indirect costs, making up only 17.6 ± 5.6%. The distribution of direct vs indirect cost proportions is shown in Figure 3.

### Cost variability

To examine cost variability, we found that 28.56% of total costs of procedures fell outside 1 SD from the mean cost. The coefficient of variation of the overall cost distribution was 0.17, the skewness was 0.73, and the kurtosis was 1.80.

### Factors associated with cost variability

In multivariable analysis, procedure operator was the factor most associated with AF ablation cost, with a difference in overall cost of approximately 12% between the lowest and highest cost operator (Table 2). In this cohort of routine, uncomplicated procedures, patient determinants of health did not appear to be associated with large variability in procedure costs to the healthcare system (<3%).

**Table 2:** Factors associated with variability in overall ablation cost to the healthcare system, expressed as average percent difference in overall cost

Variable	% Difference	95% CI
Age	–0.009	–0.112 to 0.094
Male	–1.296	–3.432 to 0.886
White	2.671	–1.679 to 7.215
Operator 1	11.961	6.033 to 18.219
Operator 2	8.647	4.336 to 13.135
Operator 3	8.026	2.708 to 13.621
Operator 4	0.166	–3.084 to 3.524
Operator 5	11.558	7.497 to 15.773
Operator 6	10.685	6.319 to 15.23
Beta-blocker	0.654	–1.506 to 2.862
Calcium-channel blocker	–0.7	–2.875 to 1.523
Prior ablation	–0.499	–2.535 to 1.579
Anticoagulation	–1.509	–5.146 to 2.267
Antiarrhythmic drugs	–0.401	–2.508 to 1.75
CHA_2_DS_2_-VASc score	–0.342	–1.008 to 0.328
Prior Cardioversion	1.477	–0.649 to 3.65
Left ventricular ejection fraction value	–0.021	–0.111 to 0.069

CHA_2_DS_2_-VASc = congestive heart failure, hypertension, age ≥75 years, diabetes mellitus, prior stroke or transient ischemic attack or thromboembolism, vascular disease, age 65-74 years, sex category; CI = confidence interval.

## Discussion

In this study, we provide the first analysis of actual costs of AF ablation procedures to a healthcare system and the components and variability of such costs. There are several important findings from this study. First, electrophysiology ablation equipment comprise the majority of AF ablation costs, followed by anesthesia support. Direct costs such as these made up over 80% of the overall costs of AF ablation procedures. Second, overall cost variability of AF ablation procedures is large, with several contributors to cost demonstrating significant variability (eg, ablation equipment, anesthesia support). Last, the procedure operator, and not the patient characteristic, was identified as the main factor associated with variability in ablation costs to the healthcare system, with a 12% range in overall average AF ablation costs between the least and most expensive operators.

It is important to consider these findings in the context of the novelty of the data presented. Previous studies have primarily assessed AF ablation payer costs; they are not a reflection of how much money it costs to deliver care.^1^^,^^3^^,^^4^ Payer costs are usually contracted and negotiated based on a variety of highly variable factors specific to the healthcare system, payer, and locale. While payer costs may be valuable for understanding population cost-effectiveness from a payer standpoint, they are less useful in informing healthcare system approaches to value-driven care. Additionally, payer costs rarely include a level of granularity to assess drivers of variability and cannot include components such as nonbilled facility resources and services. The data that we present are costs to the healthcare system for delivering the care of AF ablation based on rigorously tested and validated methodology—not theoretical estimates.^5^

Another important finding of this study is the clear majority of AF ablation costs associated with direct components that are explicitly used during an individual AF ablation procedure. Many studies have shown the exorbitant overhead required for general operating room costs that are not specific to an individual procedure.^17^ However, we have clearly shown that for AF ablations, most of the costs are direct, individual components of a procedure such as EP ablation equipment and anesthesia and not staff or other indirect resources. The likely reason for the differences in the ratio of direct vs indirect costs in the operating room vs EP lab are driven by the high cost of disposable, one-time-use devices common in the EP lab compared with multiuse devices in main operating room procedures. Our results suggest that efforts to improve cost-based value of care for AF ablation could be targeted at by reducing supplies and person-hours used during the procedure if subsequent research demonstrates that clinical outcomes are not affected by changes in cost.

One major component of procedure cost to the healthcare system was anesthesia support (∼30%)—adoption of general anesthesia for AF ablation procedures inherently increases costs. While this practice is now relatively widespread, it may be revisited in an era of emerging ablation technologies that could yield shorter procedures and potentially reduce collateral tissue injury, and with reduced monitoring requirements (eg, esophageal temperature probes).^15^ Nevertheless, there is an emerging body of evidence to support improved safety, effectiveness, and patient satisfaction for the use of general anesthesia during routine AF ablation.^18^, ^19^, ^20^

Ablation equipment was by far the predominant component of ablation costs to the healthcare system, nearly half of the overall cost per procedure. This equipment includes items such as ablation and diagnostic catheters, mapping system patches, temperature probes, and other one-time-use materials. As EP is a rapidly evolving field driven by innovations in technologies that become available nearly every year, costs for these “cutting-edge” tools, and the subsequent procedure, inevitably increase. Nevertheless, evidence supporting improved clinical outcomes for many technologies is not always consistent, with few cost-effectiveness studies performed for new ablation catheters and mapping systems, for example.^21^, ^22^, ^23^, ^24^ Further, those cost-effectiveness analyses that are performed are rarely conducted based on direct costs to a healthcare system. Given our findings that such supplies are the major contributor to healthcare system costs for these procedures, cautious adoption of newer, more expensive tools (particularly of unclear benefit) could be a prudent approach to mitigating procedural cost rises if subsequent research demonstrates clinical outcomes are not affected by changes in cost.

The overall variability of AF ablation cost was in general large. We found that 28.56% of AF ablation procedures had costs outside 1 SD from the mean cost. Additionally, the coefficient of variation, skewness, and kurtosis statistics were also high compared with what we would expect. We find this amount of variability in overall AF ablation cost high, given the selection of routine, uncomplicated, outpatient AF ablation procedures. Furthermore, >90% of procedures used the same energy source (radiofrequency) from the same mapping and catheter vendor. As such, one would expect cost variability to be low with the coefficient of variation much smaller in the 0.01 to 0.05 range. A value of 0.17 indicates that the standard deviation of the procedural cost is nearly 20% of the mean cost. In an expensive ablation procedure, this translates to large absolute cost variation.

We found that the procedure operator was associated with variability in overall AF ablation cost by up to 12%. We found that the providers with the highest AF ablation procedure cost were driven by higher costs in each category (eg, ablation equipment, imaging, pharmacy). This indicates that there is no primary driving category to explain higher cost for specific providers. Furthermore, this may be challenging to interpret and address given the pricing structures in this field. Healthcare systems receive better, preferential pricing when they use high volumes of certain ablation equipment. When operators choose to use less common ablation equipment, healthcare institutions often pay a premium price. From a healthcare system standpoint, there are 4 main ways to reduce ablation equipment costs: (1) improve consistency of supplies across operators (reduce variability in tools and make the supply chain very efficient and simple); (2) restrict available vendor selection (naturally also simplifies inventory); (3) negotiate expected price and maximum cost, allowing market forces to influence what tools are used; and (4) collaborate on purchasing across multiple centers to improve volume purchasing, similar to what is achieved in large healthcare systems. All of these approaches have strengths and weaknesses, and are common practices.

It is notable that AF ablation cost variability was not associated with characteristics of the patients—that is, older, more complex patients did not appear to significantly drive cost variability of procedures among these uncomplicated procedures. For instance, age was not associated differences in AF ablation cost variability, despite previously published links to difficulty and length of procedures.^10^^,^^11^ Only small amounts of cost differences were associated with patient characteristics (<3% of cost differences for all) compared with the large 12% differences based on the operator. The most likely reason for these small differences is due to study design, which excluded procedures with complications and those performed in hospitalized patients. Thus, the analytic cohort may be relatively healthier or homogeneous*.*^23^

### Limitations

There are limitations to our study. First, we purposefully examined routine outpatient AF ablation cases and excluded possible higher costs associated with sicker inpatient AF ablation cases, as they were not consistent with our objectives. This also complicates any interpretation of outcomes; as such, clinical outcomes are not included here. Next, this is a single-center study without data from multiple institutions. However, considering the routine nature of AF ablations and equipment used, there is a high likelihood that other systems have similar cost variability and distributions. Finally, discrete dollar values are not presented in this article for the reasons discussed previously. However, the relative numbers are informative and valuable to a broader audience because absolute costs of care to this healthcare system are influenced by local standard-of-living costs and different contracting policies and approaches. Relative cost contributions are much more likely to be externally valid and more applicable to other systems.

## Conclusion

We identified direct costs, including EP lab supply costs and anesthesia support, as making up the bulk of healthcare system AF ablation costs. We also found the cost variability of AF ablation procedures to be large. Finally, we identified the procedure operator as the main factor associated with cost variability, yielding a 12% difference in AF ablation costs between the least and most expensive operators. These data offer potential opportunities for improved efficiency of delivering AF ablation within healthcare systems.
