# Supplementary material for: Major drivers of healthcare system costs and cost variability for routine atrial fibrillation ablation
Source: Heart Rhythm O2. 2022 Dec 27;4(4):251–7. doi: 10.1016/j.hroo.2022.12.014 (PMC10134392; doi:10.1016/j.hroo.2022.12.014)
Supplement: 2022-12-15-Supplement [file mmc1.docx]

**Supplemental Figures:**

Supplemental Figure 1: Histogram distributions of the eight components of cost associated with AF ablation procedures and their relative contributions to the overall AF ablation cost.
